# Biomarkers of Subclinical Atherosclerosis in Patients with Autoimmune Disorders

**DOI:** 10.1155/2012/503942

**Published:** 2012-02-22

**Authors:** Elisabetta Profumo, Manuela Di Franco, Brigitta Buttari, Roberta Masella, Carmelina Filesi, Maria Elena Tosti, Rossana Scrivo, Antongiulio Scarno, Antonio Spadaro, Luciano Saso, Rachele Riganò

**Affiliations:** ^1^Dipartimento di Malattie Infettive, Parassitarie ed Immunomediate, Istituto Superiore di Sanità, 00161 Rome, Italy; ^2^Dipartimento di Medicina Interna e Specialità Mediche, Reumatologia, Sapienza Università di Roma, 00185 Rome, Italy; ^3^Dipartimento di Sanità Pubblica Veterinaria e Sicurezza Alimentare, Istituto Superiore di Sanità, 00161 Rome, Italy; ^4^Centro Nazionale di Epidemiologia, Sorveglianza e Promozione della Salute, Istituto Superiore di Sanità, 00161 Rome, Italy; ^5^Dipartimento di Fisiologia e Farmacologia “Vittorio Erspamer”, Sapienza Università di Roma, 00185 Rome, Italy

## Abstract

Atherosclerosis is accelerated in rheumatoid arthritis (RA) and psoriatic arthritis (PsA). We investigated a possible association of oxidized low-density lipoproteins (ox-LDLs), nitric oxide (NO), 3-nitrotyrosine, vitamin A, vitamin E, and ***β***-carotene serum levels with subclinical atherosclerosis in RA and PsA. By the use of ELISA, we observed higher ox-LDL levels in patients with intima-media thickness (IMT) > 1 than in patients with IMT ≤ 1 and a negative correlation between NO levels and IMT values. By the use of high-performance liquid chromatography, we determined higher levels of vitamin A in patients with PsA and IMT ≤ 1 than in controls and lower levels of ***β***-carotene in patients with RA and PsA than in controls. ***β***-carotene concentrations were negatively correlated to the duration of disease in RA. Our study confirms that ox-LDLs and NO may be markers of accelerated atherosclerosis in RA and PsA whereas vitamins seem to be associated only to the presence of the autoimmune disorders.

## 1. Introduction

Many studies have reported an excess of cardiovascular morbidity and mortality among patients with chronic inflammatory diseases of the musculoskeletal system such as rheumatoid arthritis RA [1]. In patients with active RA, the majority of cardiovascular deaths are caused by accelerated atherosclerosis [2–4] probably due to the chronic activation of inflammatory mechanisms. As previously reported in patients with (RA) [5], recent data have demonstrated the presence of endothelial dysfunction in patients with psoriatic arthritis (PsA) [6, 7]. The occurrence of cardiovascular disease in this pathology might be directly linked to the activation of inflammatory mediators in the skin and joints [8]. 

As RA and probably PsA predispose to atherosclerosis, an important issue is how to prevent this outcome. In the last decade, great attention has been focused on the identification of risk factors predisposing to atherosclerotic disease [9, 10] and on the associations of acute systemic inflammation markers with atherosclerosis [11, 12]. It is well known that the inflammatory overload, accompanied to the combination of an excessive production of reactive oxygen species and an impaired antioxidant defence capacity [13, 14], leads to oxidative stress in patients with rheumatic diseases and this may play an important role in driving the rheumatic disease as well as cardiovascular complications. However, the precise role of oxidative stress in the progression of these pathologies remains largely elusive. Defences against oxidative stress rely closely on vitamins, which are potent nonenzymatic antioxidants. Previous observational studies have suggested that antioxidants may protect against the development of RA, but the results for individual antioxidants are conflicting [15]. In addition, gene interactions suggest a potential contribution of inducible and endothelial nitric oxide synthase (NOS2A and NOS3) gene polymorphisms to cardiovascular event susceptibility in patients with RA [16]. The present study was undertaken to confirm our preliminary findings on ox-LDLs and NO as indicators of atherosclerosis development in patients with RA and PsA [17], as well as to investigate the possible role of serum vitamins as biomarkers of cardiovascular risk in patients with autoimmune disorders. For this purpose, we determined serum levels of ox-LDLs, NO, 3-nitrotyrosine, vitamin A, vitamin E, and **β**-carotene in patients with RA and PsA divided into two groups according to the presence or not of atherosclerotic disease determined by Echo-colour Doppler ultrasonography. As controls, we used healthy subjects free of atherosclerotic disease. We also investigated whether serum biomarkers were related to traditional risk factors for atherosclerosis or to the duration of the autoimmune disease.

## 2. Materials and Methods

### 2.1. Study Population

We enrolled 49 consecutive patients (24 with RA fulfilling the American College of Rheumatology criteria and 25 with PsA fulfilling the Classification Criteria for Psoriatic Arthritis) [18, 19] and 13 sex- and age-matched healthy subjects. Exclusion criteria for patients were (i) recent infections (<1 month), (ii) other autoimmune diseases, (iii) malignancy, (iv) inflammatory diseases before enrollment, (v) proven coronary artery disease, and (vi) diabetes mellitus or uncontrolled hypertension. All patients were taking disease modifying antirheumatic drugs at the time of enrolment. The inclusion criteria for healthy subjects were no history of myocardial infarction, coronary bypass, coronary angiography with angioplasty or stenting or both, absence of inducible coronary ischemia, demonstrated by an electrocardiogram and a color-doppler echocardiogram performed at rest and during exercise, no history of cerebrovascular accident, or peripheral vascular disease, and absence of recent infections or inflammatory diseases. Furthermore, none of healthy subjects had ultrasonographically evident carotid or femoral atherosclerotic disease. All hematological variables, including risk factors for atherosclerosis, were in the range of “normal” values. Both controls and patients who followed a standard Mediterranean diet did not take antioxidants, integrators, and/or vitamins and conducted a modest level of daily activity. Serum samples were obtained from patients and controls and stored at −80°C until use. The study protocol was approved by the local Ethics Committee and informed consent was obtained from all participants.

### 2.2. Intima-Media Thickness of the Common Carotid Arteries

The presence of atherosclerosis in patients and healthy subjects was evaluated by measurement of intima-media thickness (IMT) of the common carotid arteries. A recent meta-analysis disclosed that patients with RA have abnormally increased values of carotid IMT when compared with matched controls [20]. It was also the case in patients with PsA, even in those without classic cardiovascular risk factors [21]. Since a recent study also disclosed that a value of carotid greater than 0.90 mm predicts the development of cardiovascular events in patients with RA [22] and a carotid IMT value greater than 0.90 mm was also considered index of subclinical atherosclerosis [23], we stratified individuals into two groups according to carotid IMT values greater than 1.0 mm or equal to or lower than 1.0 mm. An IMT value > 1 was considered index of subclinical atherosclerosis.

High resolution B-mode Ultrasonography Echo-colour Doppler Images were acquired on an ultrasound system equipped with a 7.5 MHz frequency linear-array transducer.

### 2.3. Serum Levels of ox-LDLs, NO and 3-Nitrotyrosine

ox-LDLs, NO, and 3-nitrotyrosine concentrations in serum samples from patients and healthy subjects were quantified with commercially available ELISA kits (Oxidized LDL Competitive ELISA, Mercodia, Uppsala, Sweden for ox-LDL; OxiSelect Nitrotyrosine ELISA Kit, Cell Biolabs, San Diego, CA, USA; Nitrate/Nitrite Colorimetric Assay Kit, Cayman, Lausen Switzerland for NO), as recommended by the manufacturer. The detection limits of the assays were ≤0.3 U/L for ox-LDLs, <2.5 *μ*M for NO, and 1.95 nM for 3-nitrotyrosine.

### 2.4. Serum Levels of Vitamin A, Vitamin E, and *β*-Carotene

Serum levels of vitamin A, E, and **β**-carotene were determined by a high-performance liquid chromatography (HPLC) method [24]. Briefly, serum was deproteinised with ethanol containing internal standard (*α*-tocopherol acetate) and extraction of the analytes of interest was performed using hexane. Analysis was carried out using reversed-phase HPLC (kromasil 5 *μ*m, Phenomenex, Macclesfield, UK) and dual wavelength monitoring (S200, Perkin Elmer Instruments, CT, USA). A methanol-n-hexane mixture (85 : 15) was used as eluent and run at 1.0 mL min^−1^flow rate and 25°C [25].

### 2.5. Statistical Analysis

Data are expressed as medians and interquartile ranges. Mann-Whitney nonparametric test was used to investigate the significance of unpaired continuous data. Pearson's chi-squared test or Fisher's exact test, when necessary, was used to evaluate the differences in discrete baseline characteristics between groups of patients. Correlations were explored by Spearman rank correlation coefficient. *P* values less than 0.05 were considered statistically significant.

All the statistical procedures were performed by STATA 8.1 statistical package.

## 3. Results

### 3.1. Baseline and Clinical Characteristics of Patients

The main demographic characteristics of the patients enrolled are listed in the Table 1, where they are divided into two groups according to IMT values. No significant differences in the distribution of baseline and clinical characteristics were observed between patients with IMT > 1 and those with IMT ≤ 1.

### 3.2. Serum ox-LDL Levels

**Figure 1:**
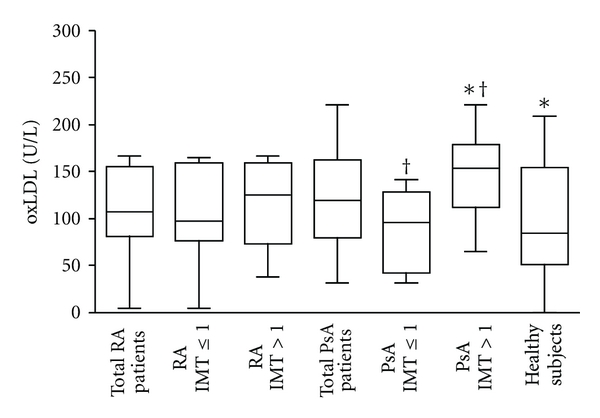
*Oxidized low-density lipoprotein (ox-LDL) concentrations in serum samples.* Box plot graphs showing ox-LDL concentrations in serum samples from the 24 patients with rheumatoid arthritis (RA) and 25 with psoriatic arthritis (PsA), divided or not according to IMT values obtained by Echo-colour Doppler images and from the 13 healthy subjects. *P = 0.049; **^†^** *P* = 0.0095.

Serum levels of ox-LDLs were higher in patients with RA and PsA than in healthy subjects (Figure 1). When patients were divided according to the IMT value obtained by Echo-colour Doppler images, these levels resulted higher in patients with IMT > 1 than in those with IMT ≤ 1 and than in healthy subjects (PsA IMT > 1 versus PsA IMT ≤ 1, *P* = 0.0095; PsA IMT > 1 versus healthy subjects, *P* = 0.049).

### 3.3. Serum NO and 3-Nitrotyrosine Levels

**Figure 2:**
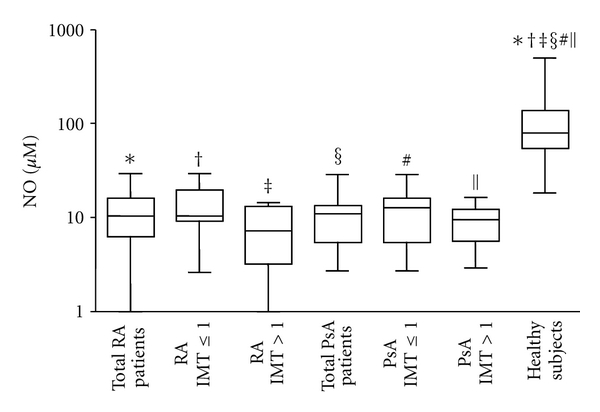
*Nitric oxide (NO) concentrations in serum samples*. Box plot graphs showing NO concentrations in serum samples from the 24 patients with rheumatoid arthritis (RA) and 25 with psoriatic arthritis (PsA), divided or not according to IMT values obtained by Echo-colour Doppler images and from the 13 healthy subjects. ^∗ **‡** § #*||*^ *P* < 0.0001; **^†^** *P* = 0.0008.

In contrast to ox-LDLs, serum levels of NO were significantly lower in patients with RA and PsA than in healthy subjects (RA and PsA versus healthy subjects *P* < 0.0001, Figure 2), especially in patients with IMT > 1. The difference between patients and healthy subjects remained statistically significant also when patients where divided according to the IMT value (RA IMT > 1 versus healthy subjects, *P* = 0.0008; RA IMT ≤ 1, PsA IMT > 1 and PsA IMT ≤ 1 versus healthy subjects, *P* < 0.0001).

**Figure 3:**
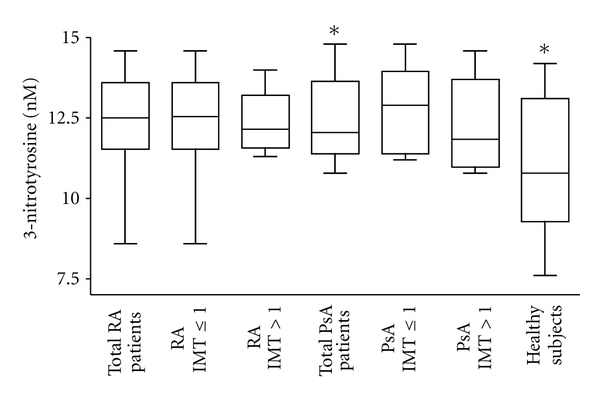
*3-Nitrotyrosine concentrations in serum samples*. Box plot graphs showing 3-nitrotyrosine concentrations in serum samples from the 24 patients with rheumatoid arthritis (RA) and the 25 patients with psoriatic arthritis (PsA), divided or not according to IMT values obtained by Echo-colour Doppler images and from the 13 healthy subjects. *P = 0.03.

To evaluate whether the significant decreased levels of NO in patients were due to a consumption of this compound in proinflammatory conditions we evaluated the modification of tyrosine residues to 3-nitrotyrosine in serum proteins. We observed that 3-nitrotyrosine levels were higher in patients than in healthy subjects (PsA versus healthy subjects *P* = 0.03, Figure 3).

### 3.4. Serum Vitamin Levels

**Figure 4:**
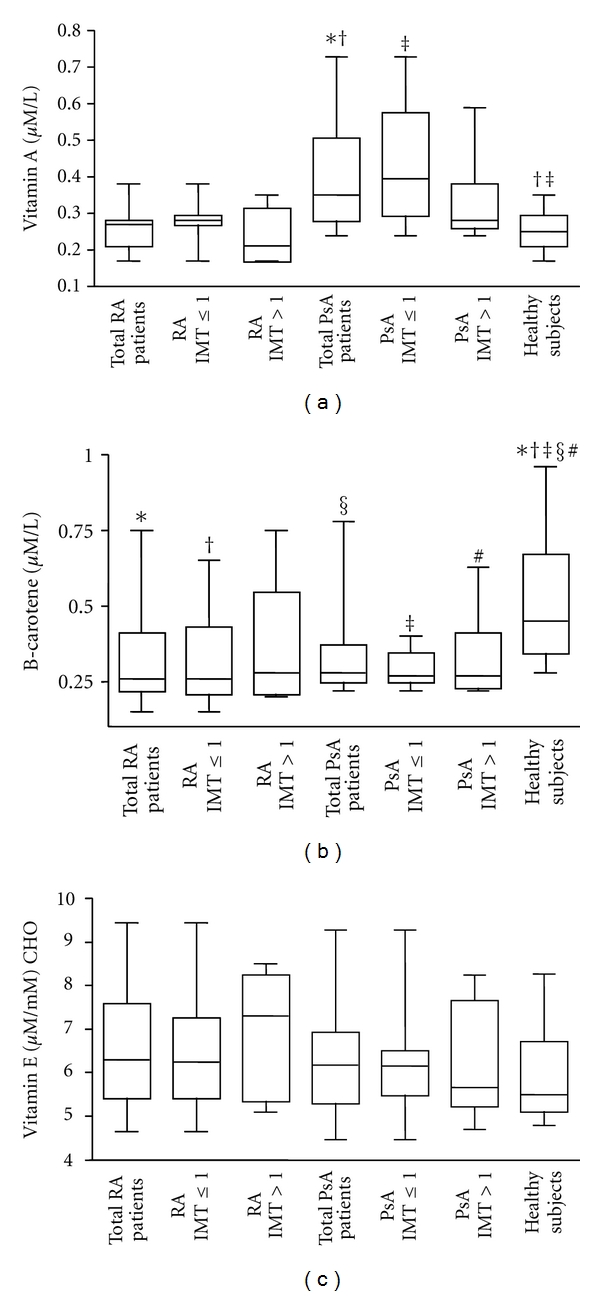
*Vitamin concentrations in serum samples*. (a) Box plot graphs showing vitamin A concentrations in serum samples from the 24 patients with rheumatoid arthritis (RA) and 25 with psoriatic arthritis (PsA), divided or not according to IMT values obtained by Echo-colour Doppler images and from the 13 healthy subjects. *P = 0.0004; ^†  ‡^ *P* = 0.0014. (b) **β**-carotene concentrations in sera. Box plot graphs showing **β**-carotene concentrations in serum samples from the 24 patients with rheumatoid arthritis (RA) and 25 with psoriatic arthritis (PsA), divided or not according to IMT values obtained by Echo-colour Doppler images and from the 13 healthy subjects. *P = 0.0021; ^§^ *P* = 0.0008; ^†^ *P* = 0.006; ^#^ *P* = 0.018; ^‡^ *P* = 0.0006. (c) Vitamin E concentrations in sera. Box plot graphs showing vitamin E concentrations in serum samples from the 24 patients with rheumatoid arthritis (RA) and 25 with psoriatic arthritis (PsA), divided or not according to IMT values obtained by Echo-colour Doppler images and from the 13 healthy subjects.

Patients with PsA showed significantly higher levels of vitamin A in serum compared to patients with RA and to healthy subjects (PsA versus RA, *P* = 0.0004; PsA versus healthy subjects, *P* = 0.0014, Figure 4(a)).When these patients were divided according to IMT values, vitamin A levels resulted higher only in patients with IMT ≤ 1 in comparison to healthy subjects (*P* = 0.0014). In PsA patients with IMT > 1, levels of vitamin A were similar to those found in RA patients and in healthy subjects.

Patients with RA and PsA showed significantly lower levels of **β**-carotene compared to healthy subjects (RA versus healthy subjects, *P* = 0.0021; PsA versus healthy subjects, *P* = 0.0008, Figure 4(b)). The difference between patients and healthy subjects remained statistically significant also when patients where divided according to the IMT value, except for RA patients with IMT > 1 (RA IMT ≤ 1 versus healthy subjects, *P* = 0.0065; PsA IMT > 1 versus healthy subjects, *P* = 0.018; PsA IMT ≤ 1 versus healthy subjects, *P* = 0.0006). No significant differences in vitamin E serum levels were observed between patients and healthy subjects or between patients divided according to IMT values (Figure 4(c)).

### 3.5. Analysis of Correlations

**Figure 5:**
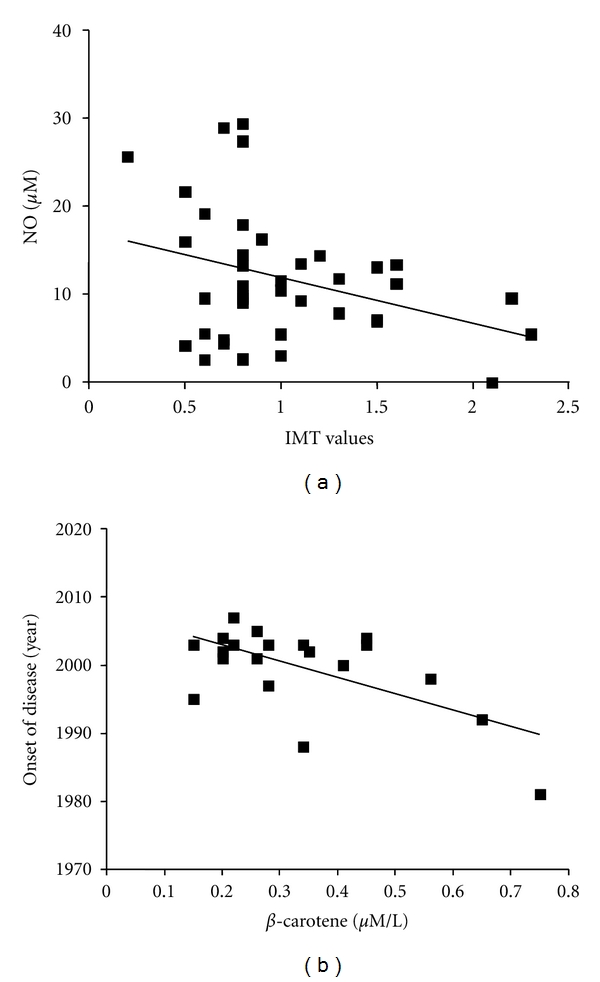
*Correlations between clinical and serological variables*. (a) Negative correlation between nitric oxide (NO) levels and intima-media thickness (IMT) values in rheumatoid arthritis (RA) and psoriatic arthritis (PsA) patients. *R* = −0.3, *P* = 0.033. (b) Negative correlation between **β**-carotene levels and the onset of the disease (evaluated in terms of year in which the disease was diagnosed) in RA patients. *R* = −0.62, *P* = 0.028.

Analysis of possible correlations among clinical and serological variables showed the presence of a positive correlation between ox-LDL serum and total cholesterol levels in patients with RA (*r* = 0.52, *P* = 0.028), and a negative correlation between NO serum levels and IMT values in patients with RA and PsA (*r* = −0.3, *P* = 0.033, Figure 5(a)). In patients with RA, a negative correlation was observed between **β**-carotene levels and the duration of disease (*r* = −0.62, *P* = 0.028, Figure 5(b)).

## 4. Discussion

The results of the present study indicate that ox-LDLs and NO may have a role as biomarkers of accelerated atherosclerosis in patients with RA and PsA. Patients with RA have an elevated risk of developing cardiovascular disease and the major underlying cause is an accelerated progression of atherosclerosis. Such a phenomenon is likely due to the fact that RA patients are exposed to chronic systemic inflammation, which is known to be a major driver of atherosclerosis. It was demonstrated that circulating markers of systemic inflammation confer a significantly higher risk of cardiovascular death among patients with RA even after controlling for traditional cardiovascular risk factors [26]. Unlike RA, only recently some studies have provided a basis for the potential association between PsA and atherosclerotic disease [7, 21, 27]. Risk factors for atherosclerosis in patients with RA and PsA include “traditional” risk factors (mainly the Framingham risk factors), as well as disease-related factors including disease duration, therapy, and inflammatory biomarkers. Most atherosclerotic risk factors accelerate disease progression by augmenting the production of reactive oxygen species (ROS). As a consequence of ROS overproduction, the cells enter a state of “oxidative stress” in which protein function in the cell is chemically modified. ROS formed during normal aerobic metabolism can also react with proteins, cholesterol, and polyunsaturated fatty acids of LDL leading to the development of oxidatively modified LDL [28]. Oxidative modification of LDL in the vascular endothelium is considered to be a key factor in the development of early atherosclerosis [29]. Our study here demonstrating higher ox-LDL serum levels in patients with RA and PsA than in healthy subjects confirms and extends previous studies reporting that ox-LDLs are raised in RA [30]. When we analyzed results on ox-LDL according to the presence or not of subclinical atherosclerosis, we observed a positive association of ox-LDL levels with intima-media thickness. In particular, PsA patients with atherosclerotic disease have ox-LDL levels significantly higher than patients without it. We can conclude that ox-LDLs, which have important proinflammatory properties, may play a role not only in RA and PsA per se but also in the chronic inflammatory condition of atherosclerosis. The association of atherosclerosis with elevated ox-LDLs support the hypothesis that chronic systemic autoimmune inflammatory process is probably a driving force for premature atherosclerosis. 

The results of the present study also showed that NO, in contrast to ox-LDLs, is decreased in sera from patients with RA and PsA, especially in those with subclinical atherosclerosis. Levels of NO in patients resulted also negatively correlated to IMT values. Serum NO levels measured by ELISA are mostly derived by iNOS induction and it is well known that during inflammation, the amount of NO produced by iNOS is increased. Previous studies reported increased endogenous NO synthesis in RA, where it contributes to T-cell dysfunction [31, 32]. Furthermore, serum levels of nitrates, nitrites, and NO_x_ (nitrites + nitrates) were found to be significantly reduced following anti-TNF-alpha therapy in RA patients [33]. The apparent contradictory findings of our study with these previous results may be explained as a consequence of NO consumption in patient sera after reaction of NO with active substances such as superoxides and consequent formation of peroxynitrites and nitrated proteins. To evaluate this possibility we have analyzed levels of 3-nitrotyrosine in protein serum samples. The presence of higher 3-nitrotyrosine serum levels in patients in comparison to healthy subjects suggests that NO reduction in patient sera may be in part due to its consumption. The small size of our population samples and the possibility that other mechanisms are involved in NO reduction may explain the lack of statistical significant differences in 3-nitrotyrosine serum levels between patients with RA and healthy subjects and the lack of correlation of these levels with IMT values.

Overall, our results on ox-LDLs and NO as biomarkers of cardiovascular risk in patients with RA and PsA strengthen our previous results [17] and are in line with findings indicating that ox-LDLs impair NO signalling and endothelial function, thus contributing to the pathogenesis of atherosclerosis [34].

In the organism, damage by ROS is counteracted with natural antioxidants and nutritional antioxidants from diet (i.e., vitamins E, C, carotenoids) [35]. Dietary antioxidants are efficient scavengers of free radicals which protect LDL against oxidation and thus reduce the oxidative damage of tissues [36]. Many factors other than dietary intake such as genetic differences in absorption or homeostatic mechanisms and environmental exposures may influence between-person variations in plasma antioxidant levels and oxidative stress [37].

It is known that antioxidants may protect against development of rheumatoid diseases by combating oxidative stress [38]. Even though an inverse relationship between systemic inflammation and antioxidant blood levels has been reported, relatively little is known about the influence of antioxidant intake on initiation of these diseases in humans [15, 38]. In particular, a lack of information exists on the relationship between antioxidants and accelerated atherosclerosis in patients with autoimmune rheumatic diseases. A question our findings help to explain is whether serum levels of vitamins, the natural antioxidants, are associated with the presence or unpresence of atherosclerotic disease in RA and PsA patients. Since the fat-soluble vitamins A, E, and **β**-carotene are essential in the regulation of autoimmune processes, the aim of the present study was to assess these vitamins in patients with RA and PsA and to find possible correlations between vitamin levels and various clinical parameters, in particular the presence or not of endothelial dysfunction. Of note, we found that in patients with PsA, vitamin A levels were significantly higher only in subjects with IMT ≤ 1. This result let us to hypothesize a possible protective effect of vitamin A on the cardiovascular system in these patients. Clearly, this interesting possibility awaits confirmation in a larger number of patients. Considering **β**-carotene, we observed that its levels were significantly lower in patients with RA and PsA than in healthy subjects but they did not differ in accordance to the presence of subclinical atherosclerosis. Our results confirm previous findings demonstrating that plasma levels of carotenoid were significantly lower in RA compared with non RA subjects and these differences remained after adjustment with potential confounders such as smoking, that is reported to affect the serum concentrations of antioxidants [39]. The same authors suggested that the reduced antioxidant concentrations found among participants with RA depend on increased metabolism of antioxidants. In line with these previous findings, our study did not disclose any associations between antioxidant levels and potential confounders among the clinical and serological variables of patients. The lower concentrations of **β**-carotene in patients in comparison with healthy subjects and mainly the inverse correlation we found between **β**-carotene content and the duration of RA suggest an association of **β**-carotene levels with autoimmune disease and not with intima-media thickness values. This result further supports the occurrence of a redox imbalance in this type of pathologies and could be connected to the higher levels of ox-LDLs found in these patients. To sustain our hypothesis, a recent report demonstrated that plasma concentrations of **β**-carotene significantly contribute to determine the occurrence of oxidative modification of LDL in vivo [29]. In that report, the authors also demonstrated that vitamin E does not influence the occurrence of ox-LDLs. In agreement with this finding, in the present study, we demonstrated unchanged vitamin E levels in the plasma of patients in comparison to healthy subjects.

The limitations of this study are mainly related to its small size, cross-sectional design, and characteristics that prevent us from determining statistical significances in some cases and from inferring any causal link between the associations reported here.

## 5. Conclusion

In conclusion, our study, although not establishing a causal link between ox-LDL or NO levels and accelerated atherosclerosis, suggests that these molecules may be operational as markers of atherosclerotic disease in patients with RA and PsA. The detection of circulating ox-LDL and NO levels in these patients might be an adjunct to imaging procedures as prognostic markers for the development of atherosclerotic disease and could be useful to the clinical management of patients. Our results also indicate that vitamins are associated only to the presence of the autoimmune disorders.

Further studies are necessary to investigate the role of vitamins in rheumatic diseases per se or in association with cardiovascular disease and to assign to these antioxidants molecules a putative role as biomarkers of cardiovascular risk in these patients.

## Figures and Tables

**Table 1:** Baseline characteristics of the 24 patients with rheumatoid arthritis (RA) and the 25 with psoriatic arthritis (PsA) divided according to intima-media thickness (IMT) values obtained by Echo-colour Doppler images.

	Patients with RA			Patients with PsA		
	IMT > 1	IMT ≤ 1	*P* ^ c^	IMT > 1	IMT ≤ 1	*P* ^ c^

*N*	6	18		11	14	
Age (years), median (range)	54 (48–65)	55.5 (22–66)	0.424	48 (35–62)	52 (35–69)	0.369
Male/female (*n*)	1/5	2/16	1.0	7/4	10/4	1.0
Duration of the disease (years), median (range)	6 (1–25)	3 (0.5–16)	0.149	4 (0.4–10)	9.5 (1–24)	0.073
Smoking^a^, *n* (%)	3 (50)	5 (28)	0.297	3 (27)	4 (28)	1.0
Hypertension^b^, *n* (%)	2 (33)	2 (11)	0.194	2 (18)	0 (0)	0.183
Total cholesterol (mg/dL), median (range)	159.5 (135–165)	155 (83–216)	0.774	170 (125–208)	179 (110–204)	0.756
HDL cholesterol (mg/dL), median (range)	34 (26–48)	43 (14–78)	0.454	31 (18–54)	33 (17–58)	0.634
Triglycerides (mg/dL), median (range)	85.5 (76–182)	79 (36–258)	0.526	103 (70–160)	98 (43–177)	0.382
ESR (mm/h) median (range)	16 (11–36)	21 (6–84)	0.449	15 (5–50)	7 (2–40)	0.088
CRP (mg/L), median (range)	5.5 (1.1−20)	2.5 (0–60)	0.716	1.35 (0–18)	2.13 (0–8.8)	0.396
Uricemia (mg/dL), median (range)	4.4 (2.9–6.2)	3.25 (2.1–8.0)	0.206	4.98 (2.9–8.7)	4.84 (2.3–11.2)	0.8

^a^Smoking is defined as current smokers;

^b^Hypertension is defined as systolic blood pressure ≥140 mmHg, diastolic blood pressure ≥90 mm Hg, or need for hypertensive medication;

^c^Pearson's chi-squared test or Fisher exact test for discrete variables and Wilcoxon test for continuous variables to evaluate the presence of statistically significant differences between RA or PsA patients with IMT > 1 and those with IMT ≤ 1;

ESR: erythrocyte sedimentation rate; CRP: C reactive protein; IMT: intima-media thickness.
